# Resveratrol Modulates the Gut-Brain Axis: Focus on Glucagon-Like Peptide-1, 5-HT, and Gut Microbiota

**DOI:** 10.3389/fnagi.2020.588044

**Published:** 2020-11-24

**Authors:** Ji Yeon Chung, Jae-Ho Jeong, Juhyun Song

**Affiliations:** ^1^Department of Neurology, Chosun University Medical School, Gwangju, South Korea; ^2^Department of Microbiology, Chonnam National University Medical School, Gwangju, South Korea; ^3^Department of Anatomy, Chonnam National University Medical School, Gwangju, South Korea

**Keywords:** resveratrol, gut–brain axis, glucagon-like peptide-1 (GLP-1), 5-hydroxytryptamine (5-HT), gut microbiota

## Abstract

Resveratrol is a natural polyphenol that has anti-aging and anti-inflammatory properties against stress condition. It is reported that resveratrol has beneficial functions in various metabolic and central nervous system (CNS) diseases, such as obesity, diabetes, depression, and dementia. Recently, many researchers have emphasized the connection between the brain and gut, called the gut–brain axis, for treating both CNS neuropathologies and gastrointestinal diseases. Based on previous findings, resveratrol is involved in glucagon-like peptide 1 (GLP-1) secreted by intestine L cells, the patterns of microbiome in the intestine, the 5-hydroxytryptamine (5-HT) level, and CNS inflammation. Here, we review recent evidences concerning the relevance and regulatory function of resveratrol in the gut–brain axis from various perspectives. Here, we highlight the necessity for further study on resveratrol's specific mechanism in the gut–brain axis. We present the potential of resveratrol as a natural therapeutic substance for treating both neuropathology and gastrointestinal dysfunction.

## Introduction

Resveratrol is a polyphenol that is secreted by grapes and berries (Wang et al., 2013) and could regulate insulin action, lipid metabolism, and glucose homeostasis (Chen et al., 2017). Resveratrol has been reported to have an anti-aging effect and to regulate inflammation in various organs (Buhrmann et al., 2017; Malaguarnera, 2019).

Recent researchers have highlighted the connection between the gut and brain, called the “gut–brain axis,” owing to the proven linkage between many factors related with the brain and the intestine (Louwies et al., 2020; Parker C. G. et al., 2020). In addition, researchers have discussed the connection between the gut and brain as key to finding therapeutic treatments for both neurological dysfunction such as cognitive decline and impaired gastrointestinal homeostasis (Rhee et al., 2009). Many cell types associated with the enteric nervous system including enteric epithelial cells, cells of Cajal, and enterochromaffin cells are influenced by the gut–brain axis (Mayer et al., 2014b).

Numerous researchers have highlighted resveratrol as a multiple regulator in various organs including the pancreas, liver, brain, and gut (Kumar et al., 2013; Movahed et al., 2013; Caron et al., 2014). Based on previous studies, we assume that there is considerable potential for resveratrol to regulate the gut–brain axis. Here, we review the significant evidences related to resveratrol's beneficial roles in the gut and brain.

## Resveratrol

Resveratrol, a natural polyphenol, is secreted by specific plants such as grapes and berries in response to stress conditions including infection, sunlight, and climate (Singh et al., 2015; De Sa Coutinho et al., 2018).

Resveratrol could boost glucose uptake in the absence of insulin (Zhao et al., 2019). It also exerts an anti-diabetic property via enhancing mitochondrial function and an anti-aging property via promoting energy expenditure (Ren et al., 2017; Zou et al., 2017). Resveratrol induces the expression of adiponectin (one of the adipokines) and improves insulin resistance in adipocytes and inhibits the inflammatory response (Sadruddin and Arora, 2009; Timmers et al., 2011). Resveratrol is specifically known to activate mammalian nicotinamide adenosine dinucleotide-dependent deacetylase SIRT1, which is involved in regulating glucose homeostasis, lipid metabolism, and the activation of mitochondrial function (Baur, 2010; Zhou et al., 2018). SIRT1 is a NAD+-dependent protein deacetylase that is a critical regulator of energy homeostasis-dependent nutrient metabolism (Vassilopoulos et al., 2011; Aguilar-Arnal et al., 2016).

Resveratrol activates mitochondrial function and enhances insulin secretion by activating SIRT1 (Ahuja et al., 2007; Ma et al., 2017). Resveratrol could also activate SIRT2, which subsequently mimics calorie restriction and expands lifespan (Smith et al., 2009; Gambini et al., 2015).

Several other studies demonstrated that resveratrol could alleviate hyperglycemia in a diabetic mouse model and obese mouse model (Ramadori et al., 2009; Rehman et al., 2018).

In the obese mouse model, some studies demonstrated that resveratrol had improved motor dysfunction, reduced fat mass, and induced positive changes in lipid profiles (Shang et al., 2008; Rivera et al., 2009; Haley et al., 2017).

Clinically, resveratrol has been reported to improve pathologies in type 2 diabetes, cardiovascular disease, and cognitive dysfunction (Novelle et al., 2015). Moreover, resveratrol could reduce fasting blood glucose and the level of HbA1c under diabetic conditions (Bhatt et al., 2012; Movahed et al., 2013).

**Figure 1:**
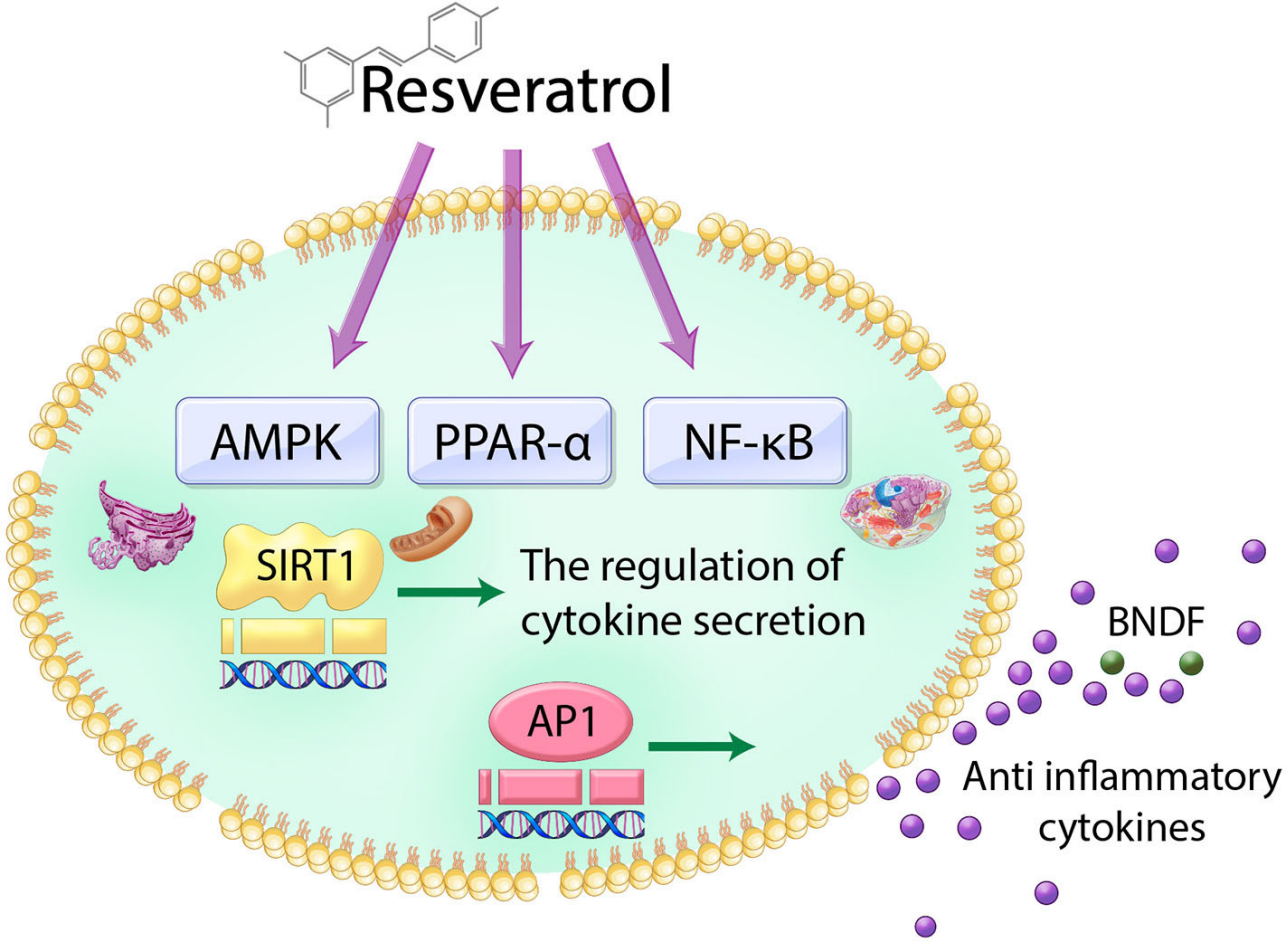
Schematic image of the action of resveratrol in cell. Resveratrol triggers the NF-κB, AMPK, and PPAR-α and subsequently regulates AP-1 and SIRT1 genes. Therefore, the action of resveratrol in the cell leads to the increase of antioxidant cytokine secretion and neurotrophic factor BDNF.

Mechanistically, resveratrol induces the secretion of insulin through sulfonylurea receptors mediated by the adenosine monophosphate (AMP)-activated protein kinase (AMPK) pathway (Hubbard et al., 2013) and peroxisomal proliferator-activated receptor α (PPARα) (Caron et al., 2014). Furthermore, resveratrol-induced SIRT1 activation attenuates inflammatory responses and pro-inflammatory cytokine secretion mainly through NF-kB- and AP-1-dependent signal pathways (Deng et al., 2008; Dao et al., 2011; Xu L. et al., 2018) (Figure 1).

In the CNS, resveratrol protects neurons which were damaged under oxidative conditions and contributed to low levels of antioxidant defense enzymes, which ultimately enhanced memory function (Kumar et al., 2013).

Similarly, another study showed that resveratrol has a neuroprotective effect via its anti-inflammatory action by regulating various neurotransmitters such as brain-derived neurotrophic factor (BDNF) and phosphodiesterases (PDEs) (Chung, 2012) (Figure 1).

Additionally, resveratrol activates the *SIRT1* gene, considered an anti-aging related gene, in the duodenum and also rescues insulin resistance and improves neuronal networks in the brain (Cote et al., 2015).

Considering these findings, resveratrol has beneficial effects against stress conditions such as inflammation, oxidative stress, hyperglycemia, and dyslipidemia. Further, resveratrol influences various organs including the brain and intestine through blood and may act as a crucial mediator in the gut–brain axis.

## Resveratrol and the GUT–Brain Axis

### Resveratrol Contributes to the Gut–Brain Axis by Regulating the Expression of GLP-1

Lately, the relationship between the gut and brain has emerged as a critical issue for treatment of neuronal disorders, such as depression and dementia, as well as gastrointestinal diseases, such as diarrhea and irritable bowel syndrome (Haj Kheder et al., 2018; Simren et al., 2018, 2019).

Based on recent researches, the pathogenesis of gastrointestinal diseases is related to the connection between the neuroendocrine network and gastrointestinal function (Koloski et al., 2012; Browning and Travagli, 2014; Yarandi et al., 2016).

Glucagon-like peptide-1 (GLP-1), an incretin hormone and a major hormone of the gut–brain axis, is linked to the control of energy homeostasis and the development of obesity (Salehi and Purnell, 2019).

GLP-1 is produced from intestinal L cells and stimulates the secretion of insulin. It enhances impaired glucose and lipid metabolism and also inhibits inflammation (Liu et al., 2013; Shah et al., 2013; Mulvihill, 2018). The major role of GLP-1 is to stimulate insulin secretion by inducing pancreatic beta cell proliferation (Morris, 2017). GLP-1 crosses the blood–brain barrier (BBB) and influences the brain as well as diverse organs (Hunter and Holscher, 2012).

In the brain, GLP-1 is synthesized by specific neurons within the nucleus of the solitary tract (Tauchi et al., 2008; Card et al., 2018). Subsequently, these GLP-1 producing neurons project to wide brain areas including the hypothalamus and cortex (Llewellyn-Smith et al., 2011; Ghosal et al., 2013).

One study demonstrated that central administration of GLP-1 leads to marked improvement of neuronal function in several brain regions such as the paraventricular nucleus, area postrema, supraoptic nucleus, arcuate nucleus, and nucleus tractus solitarius (Tauchi et al., 2008).

Another study showed that GLP-1R agonist exendin-4 could increase c-fos expression on neurons in various brain regions including nucleus tractus solitarius (Sarkar et al., 2003; Baggio et al., 2008).

GLP-1 could rapidly control glucose homeostasis after food intake, because GLP-1 receptors are located in the intestine, portal vein, pancreas, and brain, and also GLP-1 induces vagal afferent neurons innervated into gut (Iwasaki et al., 2018).

One study showed that GLP-1R antagonist's administration damages glucose tolerance and aggravates insulin resistance (Vahl et al., 2007).

In addition, another study demonstrated that the inhibition of GLP-1R in brain aggravates glucose homeostasis and insulin sensitivity (Knauf et al., 2008). Taken together, GLP-1 and GLP-1R agonist act as the important regulators of glucose homeostasis and insulin action both in systemic circulation and in CNS.

Some studies have suggested that GLP-1 has neuroprotective roles (Martin et al., 2009; Wang et al., 2018) in neurodegenerative diseases, such as Alzheimer's disease (Holscher, 2014), Parkinson's disease (Li et al., 2009), and stroke (Darsalia et al., 2012, 2018).

One study demonstrated that GLP-1 receptor signaling is considerably related to the connection between diabetes and the brain (Duarte et al., 2013). Other studies have reported that GLP-1 and its analogs including exendin-4 could protect neurons against oxidative stress in brain with dementia and dementia mimic *in vitro* cell models (An et al., 2015; Chen et al., 2016; Wang et al., 2018). Based on these findings, GLP-1 is a major gut hormone that circulates in the body and influences brain function.

One study also reported that treatment with GLP-1 receptor agonist could enhance both glycemic control and memory function (Berezin, 2016).

The positive correlation between impaired glycemic control like hyperglycemia and memory loss caused by neuronal cell damage have been proved as previous significant data (Biessels and Gispen, 2005; Carvalho et al., 2012).

Previous study mentioned that GLP-1 could be used as a controller for diabetes and ultimately GLP-1 mimic and GLP-1R agonists such as liraglutide clinically could be used for patients with diabetes in the present time (Vilsboll and Holst, 2004; Amori et al., 2007; Campbell and White, 2008; Gallwitz, 2011; Duarte et al., 2013).

Furthermore, GLP-1 may contribute to memory loss as well as improved systemic body function in diabetes by enhancing glucose tolerance and insulin resistance, suggesting that impaired glucose metabolism and poor insulin sensitivity aggravates memory loss and neuroinflammation (Rom et al., 2019).

A recent study mentioned that GLP-1R agonists improve synaptic dysfunction, strengthen long-term potentiation (LTP), and finally lead to improved cognitive function (Isacson et al., 2011).

Several studies reported that GLP-1R agonists and GLP-1 analogs could promote learning and memory function (During et al., 2003; Abbas et al., 2009), attenuate neuroinflammation against brain damage (Hattori et al., 2010), and promote neurite outgrowth, leading to stable neural connectivity (Perry et al., 2002).

One study demonstrated that GLP-1 receptor agonist exenatide could ameliorate cell stress response through SIRT1 (Xu et al., 2014).

These functions of GLP-1 receptor agonist are strongly linked to the activation of SIRT1, which could be promoted by resveratrol (Samson and Bajaj, 2013; Xu et al., 2014).

Another *in vitro* study reported that GLP-1 protects cellular apoptosis through the activation of SIRT1 in *in vitro* system (Shi and Huang, 2018).

Several studies have reported that GLP-1 and the GLP-1 receptor stimulate the activity of FoxO1 that plays a crucial role in cellular metabolism through SIRT-1-dependent FoxO1 deacetylation and Akt-dependent FoxO1 phosphorylation (Bastien-Dionne et al., 2011; Daitoku et al., 2011; Lee et al., 2012). Other studies demonstrated that SIRT1 activation by the GLP-1 agonist exendin-4 treatment protects mice under a high fat diet condition (Lee et al., 2012) and attenuates palmitate-induced ER stress and mitochondrial dysfunction (Lee et al., 2014). Resveratrol increases the release of GLP-1 in a high-fat-fed diabetes mouse model (Dao et al., 2011) and improves the epithelial cells of the intestine (Zhuang et al., 2019).

Another study demonstrated resveratrol does not directly affect the release of GLP-1 (Knop et al., 2013; Thazhath et al., 2016). Thus, the mechanism of resveratrol on the release of GLP-1 is controversial until now; we need to investigate more directly to understand the mechanism of resveratrol about the release of GLP-1.

Based on these previous studies, we assume that resveratrol could promote the effect of GLP-1 in the intestine and CNS through the activation of diverse genes such as SIRT1 and Foxo genes. Further studies on the genetic and cellular mechanisms elicited by resveratrol via GLP-1 may be helpful to understand the correlation between resveratrol and the gut–brain axis.

### Resveratrol Contributes to the Gut–Brain Axis by Involving 5-HT

Resveratrol has regulatory functions in the gut–brain axis through another hormone pathway as well as the GLP-1 pathway. Serotonin 5-hydroxytryptamine (5-HT) is expressed in both the CNS and gastrointestinal tracts, and currently 5-HT has been considered as an important target in the gut–brain axis.

5-HT is a growth factor, a paracrine factor, and an enteric neurotransmitter (Gershon and Tack, 2007), which is mainly found in the gut enterochromaffin cells. It is directly linked to depressive behavior, sleep pattern, food appetite, sexual behavior, or the control of temperature (Li et al., 2011; Yohn et al., 2017). 5-HT influences maintenance of the gastrointestinal mucosa and modulates the enteric nervous system (Gross et al., 2012).

Enterochromaffine cells in gut produce intrinsic afferent neurons of myenteric plexus in gut and are influenced by 5-HT_3_ antagonist and 5-HT_4_ agonist's inhibition (Bertrand et al., 2000; Bertrand and Bertrand, 2010; Hoffman et al., 2012). Previous studies mentioned that 5-HT produced from enterochromaffin cells in gut could promote sensory nerve activation and finally contribute to neuronal electrical activity evoked in CNS (Johanson, 2004; Chey and Cash, 2005).

Furthermore, 5-HT derived from gut protects gastrointestinal cells against neuroinflammation (Linden et al., 2005; Spohn et al., 2016).

A previous study demonstrated that the 5-HT neurotransmitter derived from brain promotes cyclic AMP (cAMP) synthesis through 5-HT receptors (Prasad et al., 2019). The change in cAMP signaling could affect both the neuropathology of major depressive disorder in CNS and gastrointestinal epithelial dysfunction in gut (Reierson et al., 2011; Cheung et al., 2019). Thus, the regulation of cAMP signaling by 5-HT should be studied further because cAMP signaling in gut and in brain contributes to various neuronal functions. Moreover, an impaired 5-HT system in gut triggers irritable bowel syndrome, and the gastrointestinal motility is increased (Grenham et al., 2011). In addition, the receptors of 5-HT have been reported to be directly involved in depression (Celada et al., 2004), anxiety, and stress-induced dyspeptic ulcers (O'mahony et al., 2006).

Considering previous data, 5-HT derived from gut and brain contributes to nervous systems globally, and the circulation of 5-HT in the body mediates the gut–brain axis (Yano et al., 2015).

A current study proved that resveratrol regulates the gut–brain axis by controlling the 5-HT-dependent pathway in an irritable bowel syndrome rat model and specifically that resveratrol influences various organs including brain hippocampus, ileum, and colon through 5-HT axis (Yu et al., 2019). One recent study highlighted that resveratrol contributes to many pathological responses through 5-HT_2C_ receptor-dependent signaling (Peng et al., 2018).

Another recent study demonstrated that resveratrol could increase the expression of 5-HT, leading to the improvement of brain function (Nabavi et al., 2017). Furthermore, the neuroprotective function of resveratrol in the depressive brain hippocampus was proved to be exerted via 5-HT (Xu et al., 2010). Most of the released 5-HT is stored in enteroendocrine cells in the intestine, and therefore gut homeostasis is important to maintain the 5-HT level in the body (Enck et al., 2016).

Several studies have mentioned the neurological role of resveratrol in depression and anxiety (Yu et al., 2013; Li et al., 2017) and the gut homeostasis-related role of resveratrol in stress-induced irritable bowel syndrome (Xu Y. et al., 2018).

One current study reported that the inhibition of 5-HT release attenuates the activation of GLP-1 receptor signaling and highlighted the relationship between GLP-1 and 5-HT serotonin system (Anderberg et al., 2017).

Another study mentioned that GLP-1 receptor agonist liraglutide could reduce the expression of 5-HT2A receptor and subsequently reduces body weight and inhibits serotonin synthesis in mice model (Nonogaki and Kaji, 2018).

Ripken et al. suggested that serotonin treatment could boost GLP-1 release, and the blocking of 5-HT receptor could affect the production of GLP-1 (Ripken et al., 2016).

A recent study proved that 5-HT enterochrnomaffin cells in gut regulates gut microbial metabolism and homeostasis and is affected by the activation of GLP-1 (Lund et al., 2018).

Further, ghrelin, known as a hormone for regulation of motivation and reward system among brain function, has been interacted with GLP-1 and the monoamine transmitter 5-HT (Currie et al., 2010; Abtahi et al., 2019).

GLP-1 derived from brain mainly is produced by the nucleus tractus solitarius in brain (Alhadeff et al., 2012). GLP-1 receptors are expressed in various brain areas including the hypothalamus, and GLP-1 projects to neurons in the ventral tegmental area, nucleus accumbens, and 5-HT-producing neurons in the dorsal raphe (Anderberg et al., 2017). Based on previous studies, the activation of GLP-1 leads to the release of 5-HT in brain, which is related with neurological behavior pattern.

Given previous evidences, resveratrol can control 5-HT and its receptor and also modulate release of 5-HT through GLP-1 regulation. Ultimately, resveratrol could control the neuropathology of neurological diseases such as depression and stress-induced anxiety. Also, resveratrol can regulate gut dysfunction in irritable bowel syndrome via 5-HT. Thus, we emphasize the necessity for further study of the specific mechanism and cellular pathways regulated by resveratrol and mediated by 5-HT to fully understand the gut–brain axis.

### Resveratrol Modulates the Gut–Brain Axis by Involving Gut Microbiota

Resveratrol is involved in the gut–brain axis through another mode in addition to the GLP-1 pathway and 5-HT system.

Recently, gut microbiota is emerging as an important node in the gut–brain axis (Louwies et al., 2020). A previous study suggested that there is an interaction between intestinal microbes and the brain and proved that intestinal microbes could dramatically enhance encephalopathy (Parker A. et al., 2020). Significant data from other studies support the function of microbiota in neurological disorders such as anxiety, autism, and depression (Mayer et al., 2014a; Sharon et al., 2019; Sun et al., 2019; Du et al., 2020).

Resveratrol administration could be metabolized by the liver, intestinal tract, and gut microbiota (Walle, 2011). A recent study demonstrated that gut microbiota contributes to metabolization of resveratrol precursors to resveratrol and also could increase resveratrol's bioavailability (Rotches-Ribalta et al., 2012; Basholli-Salihu et al., 2016). Dihydroresveratrol, 3,4′-dihydroxybibenzyl, and 3,4′-dihydroxy-*trans*-stilbene have been reported to be the major microbiota-derived metabolites made from resveratrol (Juan et al., 2010; Bird et al., 2017; Brandt et al., 2018).

Specifically, dihydroresveratrol as a metabolite of resveratrol is produced in the intestines such as the cecum, colon, and rectum through fermentation by the gut microbiota (Amri et al., 2012; Hu et al., 2019). Moreover, resveratrol was also glycosylated in the intestine to produce piceid (Rotches-Ribalta et al., 2012). Given that resveratrol was metabolized by gut microbiota (Bode et al., 2013; Carrera-Quintanar et al., 2018), resveratrol could influence the composition and diversity of gut bacteria (Carrera-Quintanar et al., 2018). Likewise, resveratrol and gut microbiota could influence each other. Specifically, *Bifidobacteria infantis* and *Lactobacillus acidophilus* are strongly linked to piceid production from resveratrol (Basholli-Salihu et al., 2016). Interestingly, a study demonstrated that resveratrol promotes gut microbiota diversity by suppressing the growth of *Enterococcus faecalis* and increasing the *Lactobacillus* and *Bifidobacterium* populations (Qiao et al., 2014).

Recently, resveratrol has been reported to improve gut microbiota in bowel diseases under harsh oxidative stress (Hu et al., 2019). One study suggested that resveratrol attenuated inflammation and improved effects of GLP-1 such as the secretion of insulin and ultimately induced a prebiotic effect to control gut microbiota in a diabetic mouse model (Dao et al., 2011).

A clinical study has reported that resveratrol treatment exerts cardiovascular and anti-obesity effects by ameliorating gut microbiota diversity (Bird et al., 2017). Resveratrol enhances the improvement of gut permeability and the integrity of intestinal tight junction proteins by controlling gut microbiota diversity (Hsieh et al., 2015; Ling et al., 2016). It has also been reported that resveratrol influences the glucuronidation and sulfation reactions in the duodenum (Wu et al., 2011).

These previous findings demonstrate that resveratrol and gut microbiota influence each other. Furthermore, resveratrol could enhance the gut microbiota diversity and the gut barrier's homeostasis. These effects of resveratrol should be investigated further to determine the specific gut bacteria that affect the gut–brain axis.

## Conclusions

**Figure 2:**
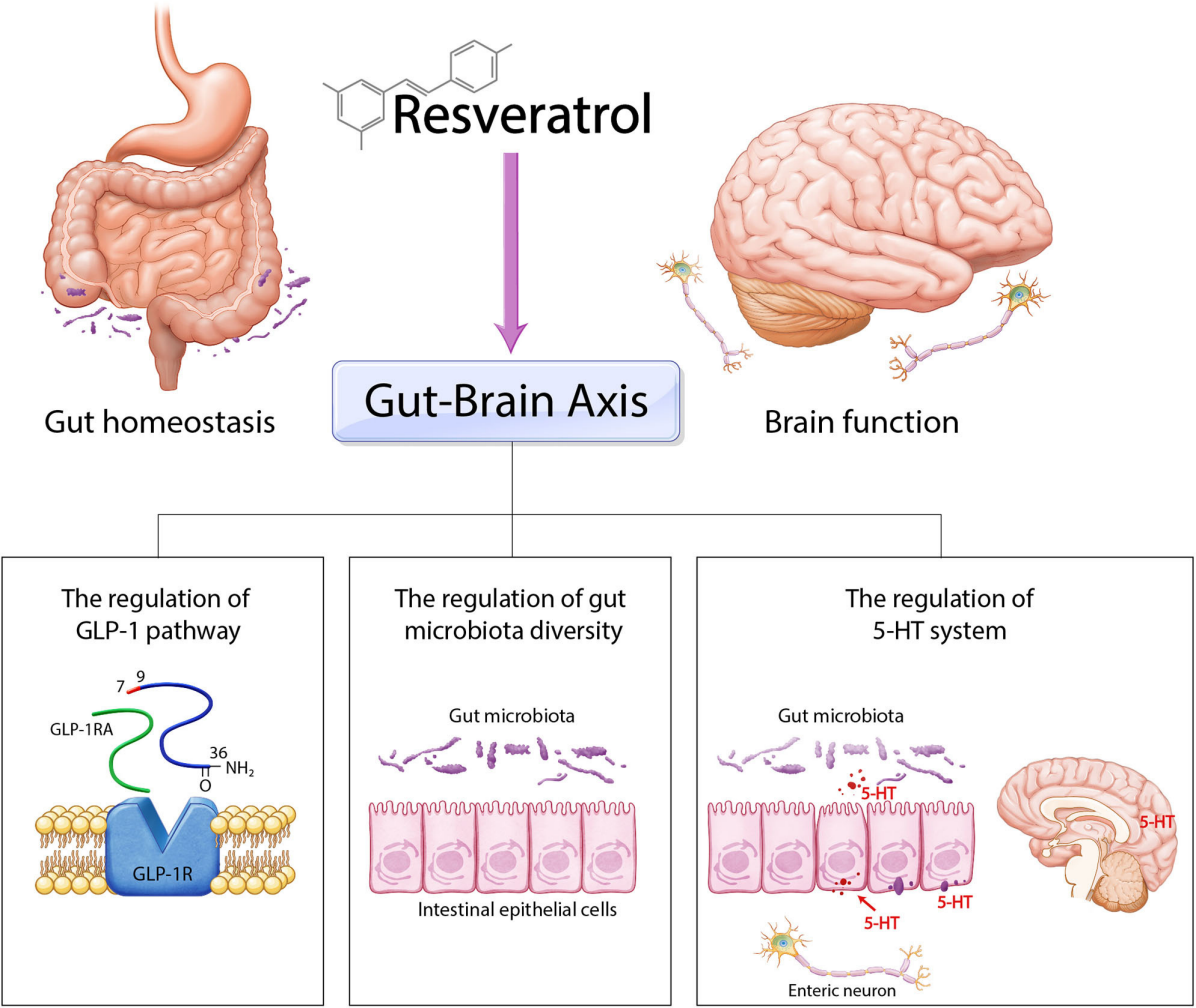
Schematic image of the role of resveratrol in gut–brain axis. Resveratrol could control gut–brain axis through three ways. Firstly, resveratrol regulates gut homeostasis and brain homeostasis by GLP-1 pathway. Secondly, resveratrol modulates gut microbiota diversity and subsequently is involved in gut–brain axis. Finally, resveratrol is related with the 5-HT system and ultimately contributes to the regulation between gut homeostasis and brain function.

Here, we reviewed previous significant evidence of the effect of resveratrol on the gut–brain axis (Table 1). We summarized three regulatory nodes of resveratrol in the gut–brain axis including the regulation of GLP-1, the involvement of the 5-HT system, and the control of gut microbiota diversity (Figure 2). Resveratrol modulates various cellular responses such as lipid droplet accumulation and insulin resistance and regulates diverse cellular signalings including AMPK, cAMP, and NF-κB signaling and also controls the balance of neurotransmitters such as BDNF and 5-HT, involved in both the progression of neuropathology and gut homeostasis. Hence, we emphasize the necessity for further experimental study about the specific mechanism of resveratrol in gut and brain. Taken together, we suggest that the application of resveratrol as a natural polyphenol for treatment of both neurological disorders and intestinal dysfunction may be a safe and effective therapeutic solution for CNS and intestinal diseases simultaneously.

**Table 1:** The relationship between resveratrol and gut–brain axis.

**Relevance between resveratrol and gut-brain axis**	**References**
**Resveratrol and gut–brain axis**	
Resveratrol and GLP-1	Bastien-Dionne et al., 2011; Daitoku et al., 2011; Dao et al., 2011; Lee et al., 2012, 2014; Samson and Bajaj, 2013; Xu et al., 2014; Shi and Huang, 2018; Zhuang et al., 2019
Resveratrol and 5-HT	Xu et al., 2010; Yu et al., 2013, 2019; Enck et al., 2016; Li et al., 2017; Nabavi et al., 2017; Peng et al., 2018; Xu L. et al., 2018
Resveratrol and gut microbiota	Dao et al., 2011; Wu et al., 2011; Amri et al., 2012; Rotches-Ribalta et al., 2012; Bode et al., 2013; Qiao et al., 2014; Hsieh et al., 2015; Basholli-Salihu et al., 2016; Ling et al., 2016; Bird et al., 2017; Carrera-Quintanar et al., 2018; Hu et al., 2019

## Author Contributions

JC, J-HJ, and JS contributed to the writing of the text. JC and J-HJ made and revised all figures. JS wrote and finalized the revised manuscript. All authors contributed to the article and approved the submitted version.

## Conflict of Interest

The authors declare that the research was conducted in the absence of any commercial or financial relationships that could be construed as a potential conflict of interest.
